# Entropy Rate Estimation for English via a Large Cognitive Experiment Using Mechanical Turk

**DOI:** 10.3390/e21121201

**Published:** 2019-12-06

**Authors:** Geng Ren, Shuntaro Takahashi, Kumiko Tanaka-Ishii

**Affiliations:** 1 Sorbonne Université, École Polytechnique Universitaire, 75005 Paris, France; 2 Graduate School of Engineering, The University of Tokyo, Tokyo 113-8654, Japan; takahashi@cl.rcast.u-tokyo.ac.jp; 3 Research Center for Advanced Science and Technology, The University of Tokyo, Tokyo 153-8904, Japan

**Keywords:** entropy rate, natural language, crowd source, Amazon Mechanical Turk, Shannon entropy

## Abstract

The entropy rate *h* of a natural language quantifies the complexity underlying the language. While recent studies have used computational approaches to estimate this rate, their results rely fundamentally on the performance of the language model used for prediction. On the other hand, in 1951, Shannon conducted a cognitive experiment to estimate the rate without the use of any such artifact. Shannon’s experiment, however, used only one subject, bringing into question the statistical validity of his value of h=1.3 bits per character for the English language entropy rate. In this study, we conducted Shannon’s experiment on a much larger scale to reevaluate the entropy rate *h* via Amazon’s Mechanical Turk, a crowd-sourcing service. The online subjects recruited through Mechanical Turk were each asked to guess the succeeding character after being given the preceding characters until obtaining the correct answer. We collected 172,954 character predictions and analyzed these predictions with a bootstrap technique. The analysis suggests that a large number of character predictions per context length, perhaps as many as $10^{3}$, would be necessary to obtain a convergent estimate of the entropy rate, and if fewer predictions are used, the resulting *h* value may be underestimated. Our final entropy estimate was h≈1.22 bits per character.

## 1. Introduction

Entropy rates *h* of natural languages have been used to investigate the complexity underlying these languages. The entropy rate of a sequence measures the amount of information per character [1] and indicates that the number of possible sequences is $2^{hn}$ for a sequence of length *n*.

Following the development of information theory and an abundance of data resources, recent studies have used computational approaches for finding the entropy rates of natural languages. Starting from the first attempt made by [2], which used a three-gram, word-level language model, various compression algorithms have been utilized [3,4]. The most recent study makes use of a state-of the art neural language model [5]. However, such computational attempts have a drawback; i.e., the computation of *h* requires a computational language model with which to predict the probability distribution of every character. As a result, the value of *h* reflects not only the complexity of the language but also the performance of the model. Indeed, in natural language processing, such an estimate of *h* is used as an indicator of the goodness-of-fit of a language model [6]. Recently reported decreases in the upper bound of *h*, for which the current minimum for English is 1.08 bpc [7] are simply highlighting improvements in the computational model.

Originally, Shannon’s study [1] and some work that followed [8,9,10,11] used cognitive methods to estimate the entropy rate *h*. The original scientific interest in *h* had to do with the complexity of human language. Given this perspective, the performance of a computational model should not be involved in obtaining a value of *h*.

The studies using cognitive approaches can be reconsidered from two perspectives. First, they were all based on limited-scale experiments. In all of these studies, a subject was asked to predict the *n*-th character given the preceding n-1 characters. According to [11], Shannon’s spouse was his only subject. Even the most recent cognitive study [11] relied on just eight subjects. Experimenting on such a small scale raises the question of the statistical validity of the acquired estimate.

Second, none of the cognitive approaches considered the limit with respect to the context length *n*. While the estimated values should be evaluated at infinite *n* by the definition of the entropy rate, the reported values are obtained at some finite *n*. In Shannon [1], the value h=1.3 bits per character (bpc) for English was obtained at n=100, and Moradi et al. [11] concluded that the estimated value does not decrease beyond n≥32 and reported a rate of h≈1.6 bpc. For extrapolation, however, a large number of observations becomes necessary in order to capture the dependence of the entropy rate on *n* well.

To that end, we conducted a large-scale cognitive test to acquire the English language entropy rate *h* through Amazon Mechanical Turk (AMT). AMT is a crowd-sourcing service offered by Amazon that allowed us to gather a large number of participants in a short time and at a reasonable cost. We focused on the entropy rate in English to make a fair comparison with Shannon [1] and other works. Other languages possibly have different values of the entropy rate, as can be seen in the comparison made in [4]. We collected a total of 172,954 character predictions from 683 different subjects. To the best of our knowledge, the scale used in this experiment was more than two times larger than any used in previous studies. At such a scale, the effects of factors that may influence the estimation of the entropy rate can be examined. Our analysis implies that Shannon’s original experiment had an insufficient sample size with which to find a convergent estimate. We finally obtained h≈1.22 bpc for English, which is smaller than Shannon’s original result of h=1.3 bpc.

## 2. Entropy Rate Estimation

### 2.1. Entropy Rate and *n*-Gram Entropy

**Definition 1.** **Shannon entropy** *Let X be a stochastic process ${\left\{X_{t}\right\}}_{t=1}^{\infty }$, where each element belongs to a finite character set X. Let $X_{i}^{j}=X_{i},X_{i+1},\ldots ,X_{j-1},X_{j}$ for i<j and $P(X_{i}^{j})$ be the probability of $X_{i}^{j}$. The Shannon entropy of a stochastic process $H(X_{1}^{n})$ is defined as*

(1) $$H\left(X_{1}^{n}\right)=-\sum_{X_{1}^{n}}P\left(X_{1}^{n}\right)\log P(X_{1}^{n}).$$

**Definition 2.** **Entropy rate** *The entropy rate h of a stochastic process X is defined as*

(2) $$h=\lim_{n\to \infty }\frac{1}{n}H\left(X_{1}^{n}\right),$$

if such a value exists [12]. The entropy rate *h* is the average amount of information per element in a sequence of infinite length.

In the following, let $F_{n}$ be the prediction complexity of $X_{n}$ given $X_{1}^{n-1}$, as follows:

(3) $$F_{n}\equiv H\left(X_{n}|X_{1}^{n-1}\right).$$

In other words, $F_{n}$ quantifies the average uncertainty of the *n*-th character given a character string with length n-1. If the stochastic process *X* is stationary, $F_{n}$ reaches the entropy rate *h* as *n* tends to infinity, as follows [12]:

(4) $$h=\lim_{n\to \infty }F_{n}.$$

In this work, *h* was estimated via $F_{n}$. A human *subject* was given $X_{1}^{n-1}$ characters and asked to predict the next character $X_{n}$. We aimed to collect a large number of predictions from many subjects. For a subject and a phrase, let a *sample* indicate the *prediction* of a $X_{n}$ given a particular $X_{1}^{n-1}$.

An *experimental session* is defined as a subject and phrase pair. For every experimental session, a subject first predicts $X_{1}$, then $X_{2}$ given $X_{1}$, then $X_{3}$ given $X_{1}^{2}$, then $X_{4}$ given $X_{1}^{3}$, …, $X_{n}$ given $X_{1}^{n-1}$, and so on. Therefore, in an experimental session, a number of observations are acquired for a given phrase, with the maximum number of observations being the character length of the phrase.

### 2.2. Shannon’s Method

If a subject guesses a character given a string of length *n*, the answer will be correct or incorrect. In Shannon’s setting and ours, the *prediction* of $X_{n}$ *by a subject is accomplished by making multiple guesses*, one character at a time, until he/she reaches the correct answer. In other words, a prediction for character $X_{n}$ in this setting consists of a series of guesses.

The number of guesses required to reach the correct answer reflects the predictability of that character and should relate to the probability of that character $X_{n}$ appearing after $X_{1}^{n-1}$. Let $q_{i}^{n}$ denote the probability that a subject requires *i* *guesses* in a prediction to find the correct letter following a block of length n-1.

Shannon deduced the following inequality [1]:

(5) $$\sum_{i=1}^{K}i\left(q_{i}^{n}-q_{i+1}^{n}\right)\log i\leq F_{n}\leq -\sum_{i=1}^{K}q_{i}^{n}\log q_{i}^{n}.$$

Here, *K* is the number of characters in the set; in this work, K=27, since the English alphabet consists of 26 letters and the space symbol. This setting corresponds to the settings used in previous works [9,11] using the cognitive approach to acquire the entropy rate in order for our results to be comparable with those reported in these works. Note that this lower bound is the lower bound of the upper bound of *h* and not the direct lower bound of *h*. For each context length *n*, the probability $q_{i}^{n}$ can be calculated for a set of samples.

In Shannon’s original experiment, 100 phrases of length 100 were taken from *Jefferson the Virginian*, a biography of ex-US President Thomas Jefferson authored by Dumas Malone. In each experimental session, the subject (i.e., only his spouse, according to [11]) was asked to predict the next character given a block of length n-1. She continued in this manner for n=1,2,…,15,and100 for each phrase; consequently, Shannon acquired 16 observations for each phrase. He used 100 different phrases; therefore, he collected 16×100= 1600 observations from his spouse in total. He then calculated $q_{i}^{n}$ for n=1,2,…,15,and100, each based on 100 observations, and the upper and lower bounds of *h* were computed based on the leftmost and rightmost terms of the inequality (5), respectively. Shannon observed a decrease in the bounds with respect to *n* and obtained an upper bound of h=1.3 bpc for n=100.

Moradi et al. [11] conducted Shannon’s experiment under two different settings. In the first experiment, they used 100 phrases of length n=64 from *Scruples II*, a romance novel authored by Judith Krantz. In the first setting, a single subject participated, and they calculated the upper bounds from n=1 to n=64 based on 100 observations. They reported that the entropy rate reached h≈1.6 bpc at n=32 and that larger values of *n* did not contribute to decreasing the upper bound. In the second setting, the eight participants were given phrases extracted from four different books, and the values of the upper bound at n=32 were reported, which ranged between h=1.62 and h=3.00 bpc.

Jamison and Jamison [9] used 50 and 40 phrases, both taken from some unspecified source, for each of two subjects, respectively. They conducted the experiment for n=4,8,12, and 100 and obtained h=1.63 and h=1.67 bpc for the two subjects at n=100 based on 50 and 40 phrase samples, respectively.

Note how the reported values deviate greatly from Shannon’s h=1.3 bpc. In all these experiments, since the number of subjects was small, the number of observations was limited, making the statistical validity questionable.

### 2.3. Cover King’s Method

While Shannon’s method only considers the likelihood of the correct answer for each $X_{n}$, Cover and King wanted to collect the distribution for each $X_{n}$. Hence, instead of counting the number of guesses required, a subject was asked to assign a probability distribution to the *n*th character given the preceding string of length n-1. Precisely, in Cover and King [10], a *prediction by a subject is the character distribution* of $X_{n}$.

They designed this experiment using a *gambling* framework, following their theory of information in gambling [13,14]. A subject assigned odds to every character which could be used for $X_{n}$; i.e., a probability distribution.

Cover and King [10] conducted two experiments separately. In the first experiment, phrases were extracted from *Jefferson the Virginian* for 12 subjects. The maximum length of a phrase was set as n=75. The estimated value of the upper bound of *h* for the 12 subjects ranged between h=1.29 bpc and h=1.90 bpc. In the second experiment, phrases were taken from *Contact: The First Four Minutes* (a science book on psychology authored by Leonard M. Zunin); lengths of n=220 were used, and two subjects participated. The estimated values of *h* produced by the two subjects were h=1.26 bpc and h=1.30 bpc.

We conducted Cover and King’s experiment using the similar framework, as explained in detail in the following section. Compared with the experiment proposed by Shannon, however, their experiment demanded too much from each subject since he/she had to set the odds for all 27 characters every time. The majority of the subjects abandoned the experiment before completing the assignment, and it was difficult to collect a large number of reliable observations. Therefore, we could not utilize this method effectively and focused on Shannon’s framework instead.

### 2.4. Summary of the Scales Used in Previous Studies

Table 1 summarizes the experimental settings of the previous reports [1,9,10,11]. We refer to the total number of observations as the sum of the count of the predictions made by the subjects for different phrases and context lengths. For example, in Shannon’s case, the total number of observations was 1600, as one subject was asked to make predictions for 16 different context lengths (i.e., n=1,2,…,15,and100) for each of 100 different phrases. The third and fourth columns in the table list the numbers of distinct subjects and phrases used in each study, respectively. Note that a phrase could be tested by multiple subjects or a subject could test multiple phrases, depending on the experimental setting.

The fifth and sixth columns present the average maximum value of *n* obtained in one session and the mean number of observations per *n*, respectively, where *n* represents the offset of a character from the beginning of a phrase. Both of these values were fixed in the previous works.

## 3. Cognitive Experiment Using Mechanical Turk

### 3.1. The Mechanical Turk Framework

Our experimental framework was implemented through Amazon Mechanical Turk, a workplace service offered by *Amazon*. AMT puts up tasks called HITs (human intelligence tasks) and *workers* do them. AMT has been used previously as a research tool for conducting large-scale investigations that require human judgment, ranging from annotating image data [15,16], to collecting text and speech data [17,18], behavioral research [19], judging music and documents [20,21], and identifying complex patterns in brain activity [22].

With AMT, the experimenter is able to collect a large number of observations on a wide range of topics. Compared with standard in-laboratory studies, however, such an experiment is open to anonymous subjects, and thus, control is limited. For example, in our case, a subject could use any external information to predict the next character. In particular, we were unable to prohibit subjects from conducting a search for the n-1 characters to obtain the answer for the next character. Furthermore, the English fluency of the subjects was unknown. Thus, the results should be examined from this perspective as well; see Section 5.2.

An experimental user interface based on Shannon’s original proposal was developed. The most important requirement of the design was the adequacy of the task load since a subject could easily lose their concentration and abandon a prediction during the experiment. We designed the user interface to be as simple as possible so as to lessen the psychological demand on the subjects.

### 3.2. Experimental Design

**Figure 1:**
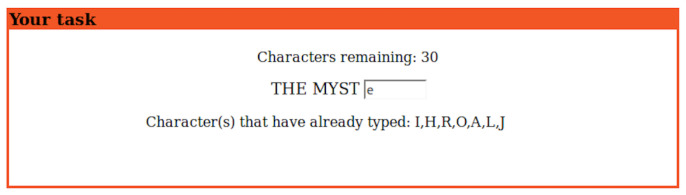
Our user interface for our cognitive experiment on Amazon Mechanical Turk. It provides: (**i**) the number of characters still available for use, (**ii**) the preceding n-1 characters, and (**iii**) the set of incorrect characters already used.

In this HIT, a subject was asked to start from the beginning fragment of a sentence, and then guess character after character of the remainder of the sentence. Figure 1 shows the interface used in the experiment. As shown, a subject received three types of information:

1. The number of characters still available for use.
2. The preceding n-1 characters.
3. The set of incorrect characters already used.

In this framework, once a subject decides on their guess, they input it and press enter to submit it. If the guess is correct, the context is updated to length *n*, and the task continues with the prediction of the n+1-th character. If the answer is incorrect, the subject must guess what the *n*-th character is until obtaining the correct answer. Subjects were informed in advance of the number of characters in the remaining phrase to avoid anyone abandoning the task.

If a phrase is too long, subjects become easily distracted. Therefore, it was necessary to adjust the length of time provided for an experimental session. Too short a time raises the cognitive load, whereas too long a time decreases a subject’s interest. After multiple trials across multiple options, such as putting a constant cap on the time allowed for each guess, we chose to allow a maximum number of guesses for every phrase. After some preliminary tests, this number was fixed to the character length of the phrase. Therefore, a subject was able to complete the task only if they always guessed all of the characters correctly. Most of the time, then, a subject was unable to finish a phrase.

The phrases were taken from the *Wall Street Journal*. In particular, 225 sentences were randomly extracted for this experiment and used as the experimental phrases. Their average length was 150.97. All characters were capitalized, and non-alphabetical symbols other than spaces were removed, duplicating the settings in previous works [1,9,10,11]. Hence, the characters were limited to the 26 letters of the alphabet, all in capital letters, and the space symbol. Table 2 lists the top ten most frequently used words and two successive words used in the experiment. As shown, they are relatively simple words that do not require specialized knowledge to predict correctly.

We considered multiple variations of Shannon’s experiment. The experiment could have consisted of guessing a character of a different phrase every time; thus, increasing the cognitive load for the subject by having them read through a different phrase every time. Another possibility was to proceed even if the character guess was incorrect. Since multiple subjects participated, it would then still be possible to acquire the probability of a correct guess. Such a method would decrease the task load substantially. However, this idea was not adopted since some subjects could choose random characters for all predictions. Finally, we reached the conclusion that Shannon’s framework was well designed and utilized it in this work.

### 3.3. Experimental Outcomes

The last row of Table 1 provides the summary for the cognitive experiment. We collected 172,954 observations from 683 different subjects, whose residences were limited to the United States, Canada, Great Britain, and Australia. The mean of the maximum values of *n* for each experimental session was 87.51. The mean number of observations collected for n≤70 was 1954.86.

These numbers are by far the largest collected for this type of experiment [1,9,10,11], in terms of both the total number of observations and the number of subjects. While these values were fixed in the previous works, they varied in our experiment due to the use of Mechanical Turk.

**Figure 2:**
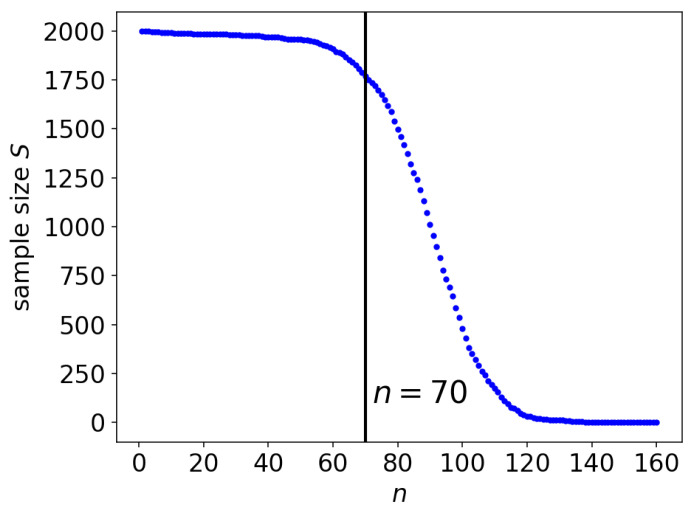
The number of observations collected for the predictions made for the *n*-th character. The vertical line indicates n=70, which provided the minimum direct estimate of $h_{expmin}=1.407$ in our experiment.

Figure 2 shows the number of samples acquired for different context lengths n-1. As the context length n-1 increased, the number of observations decreased because, in our experiment, the number of guesses could reach the maximum number of guesses allowed for a phrase, as mentioned in the previous section. For up to n=70, over 85% of the subjects made guesses. Beyond n=70, however, the number of subjects making guesses decreased quickly. As we discuss later, having a large number of observations is crucial for acquiring a good estimate of the entropy rate within a statistically reasonable margin.

### 3.4. Human Prediction Accuracy with Respect to Context Length

Shannon [1] originally reported that the upper bound decreases with respect to the context length for up to n=100. This result implies that a human is able to improve their prediction performance with more context. However, the later experiment by [11] disagreed with Shannon’s [1], as they reported that the upper bound did not decrease for n≥32. Therefore, the question remains as to whether longer contextual phrases help humans to predict future characters more accurately. Hence, we examined whether the prediction performance of subjects improved with a longer contextual phrase length, based on all observations collected.

**Figure 3:**
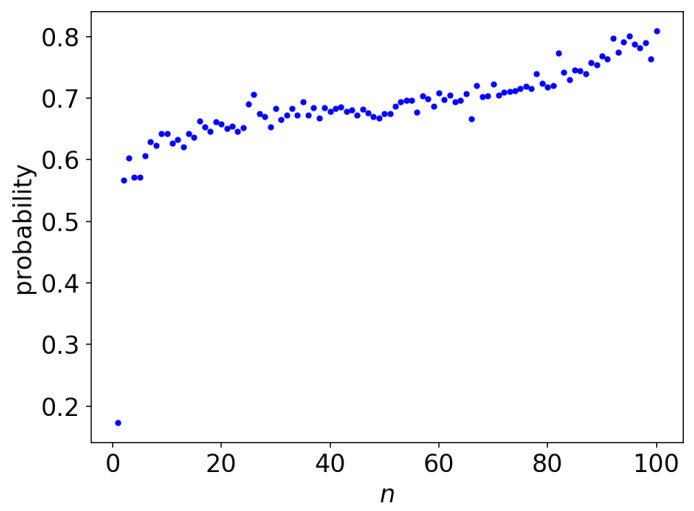
The probability that the subject needed only one guess to make the correct prediction of *n*-th character.

Figure 3 shows the probability that a subject provided the correct *n*-th character with their first guess. At n=1 (i.e., the subject was asked to predict the first character of a phrase with no context given), the probability was below 20%. The probability improved greatly from n=1 to n=2, as it reached above 50% for n=2. As *n* increased to n=100, the probability roughly monotonically increased to nearly 80%. Based on this result, a subject improves their accuracy in predicting the next character as the context length *n* increases, at least up to n=100, which supports Shannon’s claim.

This result also implies that the subjects of our experiment exhibited reasonable performances since it was a major concern that the collected observations might be of low quality due to the online experimental setting.

### 3.5. The Datapoints of the Bounds for *n*

**Figure 4:**
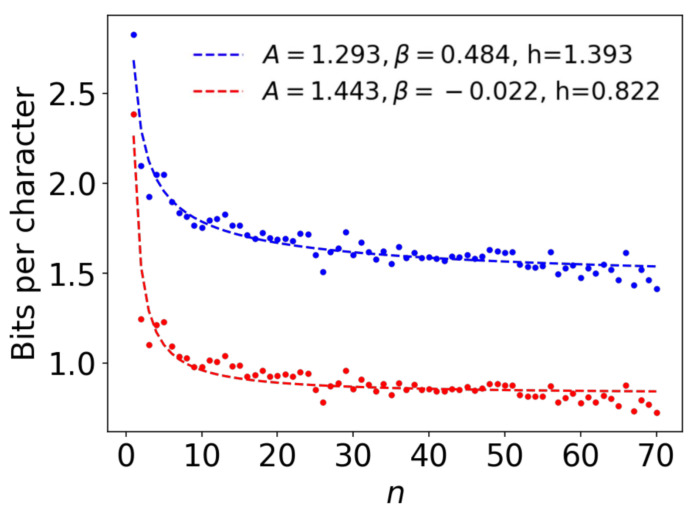
The plots of the upper bound (**blue**) and the lower bound (**red**) acquired from all observations and their extrapolations via ansatz functions of $f_{1}$ (dashed lines).

Using all of the observations, the upper and lower bounds can be estimated with Equation (5) for every *n*. The number of collected observations varies with respect to *n*, as shown in Figure 2. Figure 4 shows the plots of the upper and lower bounds computed for n=1,2,…,70 using all of the collected observations. The blue plot indicates the upper bound, whereas the red plot shows the lower bound. For the upper bound, the blue plot exhibits a decreasing tendency, although the values fluctuate along with *n*. Our main interest lies in the upper bound.

Plots of both bounds have large fluctuations for n>70 due to the decrease in the sample size for large *n*, which will be examined later in Section 5.1. The minimum experimental value of the upper bound was $h_{expmin}\equiv 1.407$ bpc, which was located at n=70. Since this is the minimum of the direct experimental values, any computed entropy rate larger than this would appear to be invalid. In the remainder of this paper, the observations collected up to n=70 are utilized.

## 4. Extrapolation of the Bounds with an Ansatz Function

As mentioned in the Introduction, the other drawback of the previous studies utilizing the cognitive approach to the entropy rate lies in not extrapolating the experimental values. Precisely, in the previous cognitive experiments [1,10,11], the reported entropy rate values were the direct upper bounds at the largest *n* used, such as n=100 in [1].

As the entropy rate, by definition, is the value of $F_{n}$ with *n* tending to infinity, its upper and lower bounds, as *n* tends to infinity, must be considered and can be examined via some extrapolation functions.

### 4.1. Ansatz Functions

As the mathematical nature of a natural language time series is unknown, such a function can only be an ansatz function. The first ansatz function was proposed by Hilberg [23], who hypothesized that the entropy rate decreases according to the power function with respect to *n* based on the experimental results of Shannon [1]. This function is as follows:

(6) $$f_{1}\left(n\right)=An^{\beta -1}+h,\;\beta <1.$$

Originally, this function was proposed without the *h* term. There have been theoretical arguments as to whether h=0 [2,3,4,5,7,24,25]; therefore, a function with the *h* term was considered in this work.

Takahira et al. [4] suggested another possibility that modifies the function $f_{1}\left(n\right)$ slightly, which is as follows:

(7) $$f_{2}\left(n\right)=\exp\left(An^{\beta -1}+h\right),\;\beta <1.$$

They observed that the stretched exponential function $f_{2}\left(n\right)$ leads to a smaller value of *h* by roughly 0.2 bpc in a compression experiment for English characters.

Schümann and Grassberger [3] introduced another function $f_{3}\left(n\right)$ based on their experimental result:

(8) $$f_{3}\left(n\right)=An^{\beta -1}\log n+h,\;\beta <1.$$

These three ansatz functions $f_{1}$, $f_{2}$, and $f_{3}$ will be evaluated based on their fit to the data points discussed in the previous section. For $f_{1}$ and $f_{3}$, *h* is the estimated value at infinite *n*, whereas in the case of $f_{2}$, the estimated value of the upper and lower bounds at infinity is $e^{h}$.

### 4.2. Comparison among Ansatz Functions Using All Estimates

**Figure A1:**
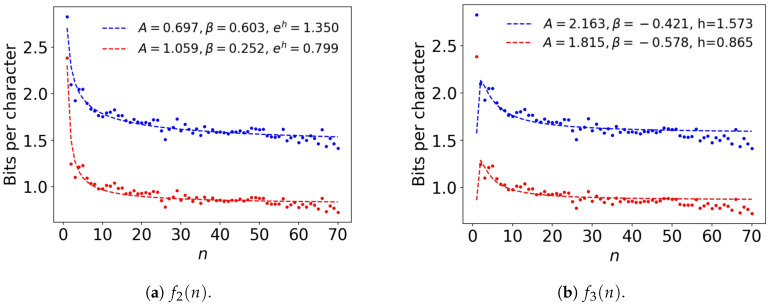
The plots of the upper bounds (**blue**) and lower bounds (**red**) acquired from all observations and their extrapolations via the ansatz functions $f_{2}$ and $f_{3}$ (shown as the dashed lines)..

Every ansatz function was fitted to the plots of the upper and lower bounds via the Levenberg–Marquardt algorithm for minimizing the square error. The ansatz functions’ fits to the data points mentioned in Section 3.5, are shown in Figure 4 for $f_{1}$ and in Figure A1 in the Appendix A for $f_{2}$ and $f_{3}$.

For $f_{1}$ and $f_{2}$, the fits converged well and the errors were also moderate. The mean-root-square error of $f_{1}$ was 0.044, quite close to the error of $f_{2}$, which was 0.043. Both the entropy rate estimates also converged to similar values of *h*; namely, h=1.393 and h=1.353 bpc, respectively, for the upper bounds. The values of β, were 0.484 and 0.603 for $f_{1}$ and $f_{2}$, respectively, suggesting monotonic decay in both cases.

On the other hand, $f_{3}$ presented some problems. The function did not fit well, and the error was 0.069. Above all, $f_{3}$’s extrapolated upper bound was h=1.573 bpc. The value is larger than the minimum experimental value $h_{expmin}=1.407$ bpc considered in Section 3.5.

This tendency of $f_{3}$ to overestimate the value *h* may be the result of $f_{3}\left(n\right)$ having been designed based on the convergence of the entropy rate of some random sequence. Therefore, a suitable ansatz function would be either $f_{1}$ or $f_{2}$. As seen, they provide similar results, which is consistent with the original observation provided in [4]. Consequently, we focus on $f_{1}$, the most conventional ansatz, in the following section.

## 5. Analysis via the Bootstrap Technique

Section 2.3 mentioned that the scale of our experiment was significantly larger than the scales used in previous experiments [1,9,11]. The large number of observations allowed us to investigate the effect of the number of observations via the bootstrap technique, which uses subsets of the experimental samples.

### 5.1. The Effect of the Sample Size

*B* sets of observations, each of which include *S* records of the experimental sessions, were sampled without redundancy. Let *S* be referred to as the *sample size* in the following discussion. As defined in Section 2.1, a record of an experimental session consists of a series of the number of guesses for each context of length n-1 produced by the same subject for a phase.

For each set, the upper bound of every *n* is the rightmost term in Equation (5), and an acquired set of points is extrapolated with the ansatz function $f_{1}$. We obtain *B* different values of *h*. In addition to their mean value, it would be reasonable to examine the interval between some bounds for the entropy rate estimate. We consider these bounds based on the fixed percentile of *B* values of *h*. We set B=1000 and acquired the means and both bounds at 5% upper/lower percentiles for different values of *S*.

**Figure 5:**
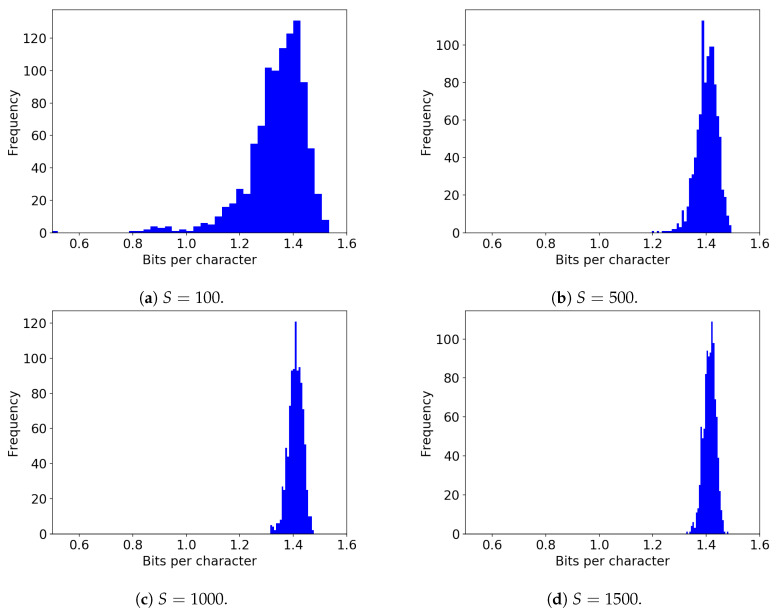
Histograms for the estimated values of the upper bound of the entropy rate h for different sample sizes. (**a**) S=100; (**b**) S=500; (**c**) S=1000; (**d**) S=1500.

Figure 5 shows the histograms of *h* values for S=100,500,1000, and 1500. At S=100, the estimated values vary widely, and the 5% percentile bounds are h=1.124 bpc and h=1.467 bpc, as shown in Table 3. The previous experiments, including Shannon’s study [1,9,11], used a maximum of S=100 observations for certain values of *n*. Our results suggest that the values reported by these works have large intervals around them and should not be considered to be general results.

Furthermore, for small *S*, the estimated values also tend to be biased towards smaller values. The mean value at S=100 was h=1.340 bpc, which is about 0.07 bpc smaller than the value h=1.412 bpc obtained for S=1000. This underestimation occurred due to the fact that an event with small probability cannot be sampled when the sample size is small. Such events with small probabilities then contribute to increasing the entropy. When their contributions are ignored, the estimate tends to be smaller than its true value. Consequently, Shannon’s original experiment could have underestimated the upper bound.

**Figure 6:**
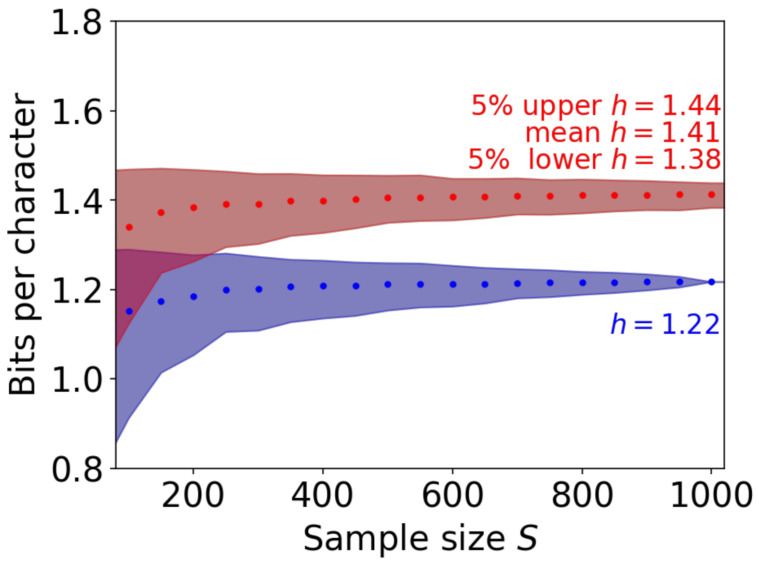
The estimated upper bounds with ansatz function $f_{1}$ using: (1) 1000 experimental sessions with the best prediction performances (**blue**), and (2) all experimental sessions (**red**), with the values reported in Table 3. The blue and red points indicate the mean values for the B=1000 sets, and the shaded areas indicate the 5% percentile bounds.

These observations suggest that a large sample size is necessary to obtain convergence of the upper bound. As observed in the values reported in Table 3, the histograms Figure 5, the red data points, and the shaded area in Figure 6, the differences between the 5% upper/lower percentile bounds decrease with larger sample size *S*. At S=1000, the difference between the bounds is smaller than 0.1 bpc, which is a reasonably acceptable margin of error.

### 5.2. The Effect of Variation on Subjects’ Estimation Performances

Our experiment was conducted with anonymous subjects, and therefore, was less controlled than an in-laboratory experiment. Such factors could influence the entropy rate estimate; therefore, the bias is examined in this section.

Although the residences of the participants were limited to native English speaking countries, as mentioned in Section 3.3, we could not control the native tongues of our participants. Although our phrases were extracted from the *Wall Street Journal* and the terms and expressions were easy to understand, even for non-natives (see Table 2), the results might be biased. In addition, the experiment was not supervised on site; therefore, subject conditions could have varied.

In principle, the entropy rate measures the maximal predictability of the text. Therefore, each estimated value should be obtained based on the maximal performance of the subject. Here, we consider estimating the entropy rate with only the best-performed experimental sessions. We first defined the performance of an experimental session as the average number of guesses required to predict the succeeding character $X_{n}$. The experimental sessions for which the maximal *n* was less than 70 were filtered out in order to keep the sample size the same for all n=1…70.

Next, the experimental sessions were sorted by performance, and the S=1000 best sessions are selected. Note that this *S* was necessary for obtaining convergence, as seen in the previous section.

We evaluated the mean and 5% percentile bounds of the best-performing set by measuring the upper bound *h* from B=1000 sets of S=100,150,200,…,1000 sub-samples. At S=1000, there is only one possible set; therefore, *h* can have just one value. The results are shown in Figure 6. The blue data points in the middle show the means, and the blue-colored areas around them shows the intervals contained within the 5% percentile bounds. Similar to the results for all experiment sessions (shown as red data points and a red-shaded area), the widths of the intervals are quite large for small sample sizes, such as S=100, and decrease towards S=1000. The mean value of the upper bound increased with respect to *S*, which is also similar to the result for all experiment sessions.

Using just the selected experimental sessions, the final estimated value converged to h≈1.22 bpc, which is smaller than the value estimated when using all experimental sessions $h_{expmin}$ and those acquired by previous cognitive experiments.

## 6. Discussion

### 6.1. Computational versus Cognitive Methods

In parallel with the cognitive approach, computational approaches have also attempted to estimate the entropy rate’s upper bound for natural language. Such an approach requires that some language model be used, and previous estimates have been found with, for example, the *n*-gram language model [2], compression algorithm model [3,4], and neural language model [5,7]. In particular, Brown et al. [2] constructed the word-level *n*-gram language model and obtained h=1.63 bpc, whereas Takahira et al. [4] conducted a compression experiment using giga byte-scale newspaper corpora and obtained an estimate of h=1.32.

In addition to the compression algorithms and *n*-gram language models, recent works have also employed neural language models, which potentially have higher capacities for accurately predicting future characters. Recently, Dai et al. [7] reported h=1.08 bpc when using Transformer XL on text8. This dataset is a collection of natural language text taken from Wikipedia and cleaned to the point of having only 26 alphabet characters and space corresponding to the setting of the Shannon’s experiment. That *h* value was smaller than our estimated value, suggesting that humans may not be able to outperform computational models in character guessing games. Nevertheless, it is worth considering the differences in the conditions of the experiments.

The primary factor is the context length. Dai et al. [7]’s model utilized several hundred context lengths to acquire their results. The high performance of the neural language models can be explained, at least partially, by their ability to utilize long contexts. However, humans *are* also able to utilize long contexts, at least as long as $n\approx 10^{2}$, to improve their prediction performances, whereas our experiment used the context lengths of up to n=70 to obtain the upper bound for *h*.

Furthermore, while a cognitive experiment obtains the upper bound of the entropy rate from the number of guesses, when using the computational model, the estimate is calculated based on the probability assigned to the correct character. With a distribution at hand, the upper bound of the computational model can be evaluated more tightly and precisely. The design of an experiment that incorporates a longer context length and character probability distributions is a direction of research that may be pursued in future work.

### 6.2. Application to Other Languages and Words

This work focused on English, which is the most studied language within the context of entropy rate estimation. Shannon’s experiment is applicable to other languages if the alphabet size of the writing system is comparable with that of English.

In contrast, for ideographic languages such as Chinese and Japanese, which have much larger alphabet sizes, it is practically impossible to conduct Shannon’s experiment. A prediction could involve thousands of trials until a subject reaches the correct character. Therefore, a new experimental design is required to estimate the entropy rate for these languages with large alphabet sizes.

Such an experimental setting would be also applicable to the estimation of the entropy rate at the word level, which could be interesting to investigate via a cognitive approach. Humans partly generate text word by word and character by character (sound by sound). Thus, any analysis could reveal new information about linguistic communication channels, including their distortions, as studied in [26,27].

### 6.3. Nature of *h* Revealed by Cognitive Experimentation

Provided with some previous work and the good fit of an ansatz extrapolation function while assuming that h≥0 and using what we consider reliable data points, we arrived at *h* = 1.22.

There is more than one way, however, to investigate the true value of *h*. Figure 4 shows how data points for larger *n* become lower than the estimated ansatz, perhaps suggesting that the values tend to zero even for larger *n*. It could be the case that *h* goes to zero. Indeed, a function without an *h* term (i.e., h=0) would fit reasonably well if the upper bound is evaluated only with relatively small datapoints of *n* such as n≤70. Overall, our analysis does not rule out the possibility of the zero entropy rate.

One observation gained from this work that highlighted the sample size is that data points are distributed and statistical margins must be considered. Hence, *h* should be considered as having a distribution and not as a single value. One such way of analysis was described in Section 5.

## 7. Conclusions

This paper presented a large-scale cognitive experiment for estimating the entropy rate for English. Using AMT, we conducted Shannon’s experiment online and collected 172,954 character predictions in total across 683 subjects. It was by far the largest cognitive experiment conducted thus far, and the scale enabled us to analyze the factors that influence the estimation.

While Shannon implied that subjects’ prediction performances improved with increasing context length, others disagreed with his implication. Our experiment showed that subjects’ prediction performances improved consistently with increasing context length, at least up to 100 characters.

Further, we investigated the influence of the number of observations on the estimation via the bootstrap technique. One of the most important insights gained is that the number of prediction observations must be at least 1000 in order to produce an estimate with a reasonable margin of error. In the case of small samples, the value of *h* could be potentially underestimated. Hence, Shannon’s original experiment and other previous experiments provided estimates that could have been underestimated. We believe that this present work reports a statistically reliable estimate with a reasonable margin of error.

Due to the online environment, the performances of the subjects varied, and the upper bound should be evaluated based on filtered results. With a sufficient number of well-performing samples, we obtained an upper bound of h≈1.22 bpc, which is slightly smaller than Shannon’s reported value of h=1.3 bpc.

Future work could include finding a new experimental design, one in which the participants use longer contexts to predict the next character; thus, reducing the cognitive load. Such an experiment would contribute to the tighter evaluation of the upper bound of the entropy rate. It would be also interesting to examine the entropy rates of other languages and at the word level while still utilizing a cognitive experiment. 

## Figures and Tables

**Table 1:** Comparison of the scales of cognitive experiments undertaken in previous works for the entropy rate estimation in English [1,9,10,11] and that of the present work.

	Total Number	Number of	Number of	Max *n*	Number of
	of Samples	Subjects	Phrases	for a Session	Sample Per *n*
Shannon [1]	1600	1	100	100	100
Jamison and Jamison [9]	360	2	50 and 40	100	50 and 40
Cover and King [10] No.1	440	2	1	220	2
Cover and King [10] No.2	900	12	1	75	12
Moradi et al. [11] No.1	6400	1	100	64	100
Moradi et al. [11] No.2	3200	8	400	32	100
Our Experiment	172,954	683	225	87.51	1954.86

**Table 2:** The top ten most frequently used words along with two subsequent words appearing in the phrases used in our experiment.

Rank	Word	Frequency	Two Subsequent Words	Frequency
1	market	15	interest rates	4
2	company	13	future contracts	3
3	investment	11	program trading	3
4	price	11	stock market	3
5	people	11	money managers	3
6	companies	10	same time	2
7	stock	9	wide variety	2
8	buy	9	time around	2
9	officials	7	higher dividends	2
10	growth	7	some firms	2

**Table 3:** The means and the 5% percentile-bound-intervals for the upper bound of *h* found by using the ansatz function $f_{1}$ for S=100, 500, 1000, and 1500. The number of sets is B=1000. The error is large for a small sample sizes, such as S=100, as the difference between the 5% percentile upper and lower bounds is larger than 0.3 bpc. This difference decreases with increasing *S* and eventually becomes smaller than ±0.1 bpc for S≥1000.

Sample Size *S*	Mean	5% Upper	5% Lower
100	1.340	1.467	1.124
200	1.383	1.468	1.263
300	1.391	1.459	1.302
400	1.398	1.456	1.327
500	1.405	1.455	1.349
1000	1.412	1.438	1.383
1500	1.411	1.444	1.374

## Appendix A

The fits of $f_{2}$ and $f_{3}$ to the same data points (as opposed to $f_{1}$, shown in Figure 4) are shown in Figure A1.
